# Playgrounds as microbial interfaces: strategies to enhance soil microbiomes and support healthy childhoods

**DOI:** 10.1128/msystems.01662-25

**Published:** 2026-03-09

**Authors:** Juulia Manninen, Aku Korhonen, Karen L. Johnson, Outi Tahvonen, Anna Luukkonen, Mika Saarenpää, Riikka Puhakka, Laura Uimonen, Laura Kummola, Chrysanthi Skevaki, Harald Renz, Juho Rajaniemi, Olli H. Laitinen, Marja I. Roslund

**Affiliations:** 1 Natural Resources Institute Finland Luke 419837, Helsinki, Finland; 2 Ecosystems and Environment Research Programme, Faculty of Biological and Environmental Sciences, University of Helsinki 89214 , Lahti, Finland; 3 Department of Engineering, Durham University 537944, Durham, United Kingdom; 4 HAMK Bio Research Unit, Häme University of Applied Sciences 3700, Hämeenlinna, Finland; 5 Faculty of Health Care and Social Services, LAB University of Applied Sciences 4371, Lahti, Finland; 6 Faculty of Built Environment, Tampere University 205520, Tampere, Finland; 7 Faculty of Medicine and Health Technology, Tampere University 101287, Tampere, Finland; 8 Institute of Laboratory Medicine, Universities of Giessen and Marburg Lung Center (UGMLC), Philipps Universität Marburg, German Center for Lung Research (DZL) 9377, Marburg, Germany; 9 Department of Environmental Health, Harvard T.H. Chan School of Public Health 218854 , Boston, Massachusetts, USA; 10 Centre for Research and Education in Public Health (CEPHRE), Academy of Athens 69100, Athens, Greece; 11 Department of Paediatrics, Kilimanjaro Christian Medical Centre University (KCMCU) 108094, Moshi, Tanzania; 12 Tongji Medical School, Shanghai, China; The University of Maine, Orono, Maine, USA

**Keywords:** microbiome, planetary health, children, human microbiome, host response, environmental microbiology, biodiversity, immune regulation, ecosystem services, multi-omics

## Abstract

Emerging evidence suggests that reduced exposure to biodiversity, including rich environmental microbiota, is associated with negative outcomes in the health and well-being of children. Biodiversity loss not only impacts individual health but also poses significant threats to planetary health. It destabilizes systems that regulate climate, purify air and water, maintain soil fertility, and support plant and microbial life essential for environmental health. Here, we review the scientific evidence on microbiome-supportive strategies in eco-centric, child-friendly playground environments. Investigating how environmental features influence soil microbiomes and exposure pathways could provide insights into how playgrounds function as living interfaces. These are places where environmental microbes shape children’s microbial colonization patterns, immune and endocrine regulatory systems, while also contributing to ecosystem services such as biodiversity support and pollutant mitigation—particularly relevant given that many pollutants are known to disrupt immune and endocrine functions in children. These dynamics have far-reaching implications for child well-being, preventive health strategies, physical activity, environmental literacy, and broader sustainability. A multi-omic systems approach offers a critical pathway to uncover the ecological and health-related impacts of nature-associated microbial exposure and characterize host–microbiome interactions underlying immune and endocrine regulation, brain development, cognition, and stress-related disorders. Our review highlights a lack of such integrative studies, underscoring the need to advance this line of research to inform evidence-based, sustainable, and health-promoting urban design.

## INTRODUCTION

Playgrounds are important outdoor environments during childhood that offer a unique opportunity to positively influence the development of children, both physiologically and psychologically. The role of playgrounds in promoting children’s health has mostly focused on physical activity, followed by social and mental health, motor skills, and weight status (1). Nowadays, it is increasingly recognized that exposure to a rich environmental microbiota in early childhood plays an important role in health and development, including the immune and endocrine systems (2–5). Despite this, only a few studies have explored how playgrounds can promote child microbial exposure and immune regulation (6–8). In response, frameworks have been proposed for microbiome-informed planning in early childhood environments (9). However, there is a lack of research addressing the broader role of biodiverse soil microbiota in supporting both environmental microbial functions related to key ecosystem services and children’s commensal microbiota associated with immune and endocrine regulation. As a result, most playgrounds and urban green spaces still fall short of integrating beneficial microbial functions into their design and often do not align with the broader goals of planetary health.

An important element in the planetary health context is soil, the balanced functioning of which largely depends on the soil microbiome (10). The soil microbiome governs many functions in the biosphere, providing a wide range of provisioning, regulating, and supporting ecosystem services that are essential for human health and well-being (11–13). A key hotspot of microbial activity is the rhizosphere, where plants and microbes interact to mobilize nutrients, suppress pathogens, and enhance stress tolerance (14). These interactions underpin vital ecosystem services, including carbon sequestration and pollutant mitigation, which are relevant to eco- and child-friendly green spaces. Today, however, many urban playgrounds are dominated by artificial turf or sealed surfaces. These surfaces may disrupt microbial-driven ecosystem services and diminish both child health benefits and ecological functions such as carbon storage and pollutant mitigation.

Soil and vegetation host diverse microbial communities, including soil- and plant-associated endophytic, rhizosphere, and phyllosphere microbiota, which can act as sources of beneficial microbes (15–18). Regular contact with these microbes, through playing, touching plants, or gardening activities, may help shape the gut and skin microbiota, contributing to immune system development and resilience (6–8, 16, 19). Designing and maintaining green spaces that maximize microbial diversity could, therefore, provide important health benefits for children.

Overall, we have a good understanding that interacting with nature promotes children’s physical health and also their psychological well-being, social behavior, stress reduction, and cognitive performance (20, 21). At the same time, the gut microbiome of children is increasingly recognized as a regulator of stress, behavior, and cognitive development (22–25), suggesting a potential link between nature exposure and gut microbiota and psychological outcomes via gut–brain axis. Positioning playgrounds as microbial interfaces, therefore, shifts the focus of design beyond physical activity and safety, toward fostering environments that also nurture beneficial host–microbe interactions.

**Fig 1:**
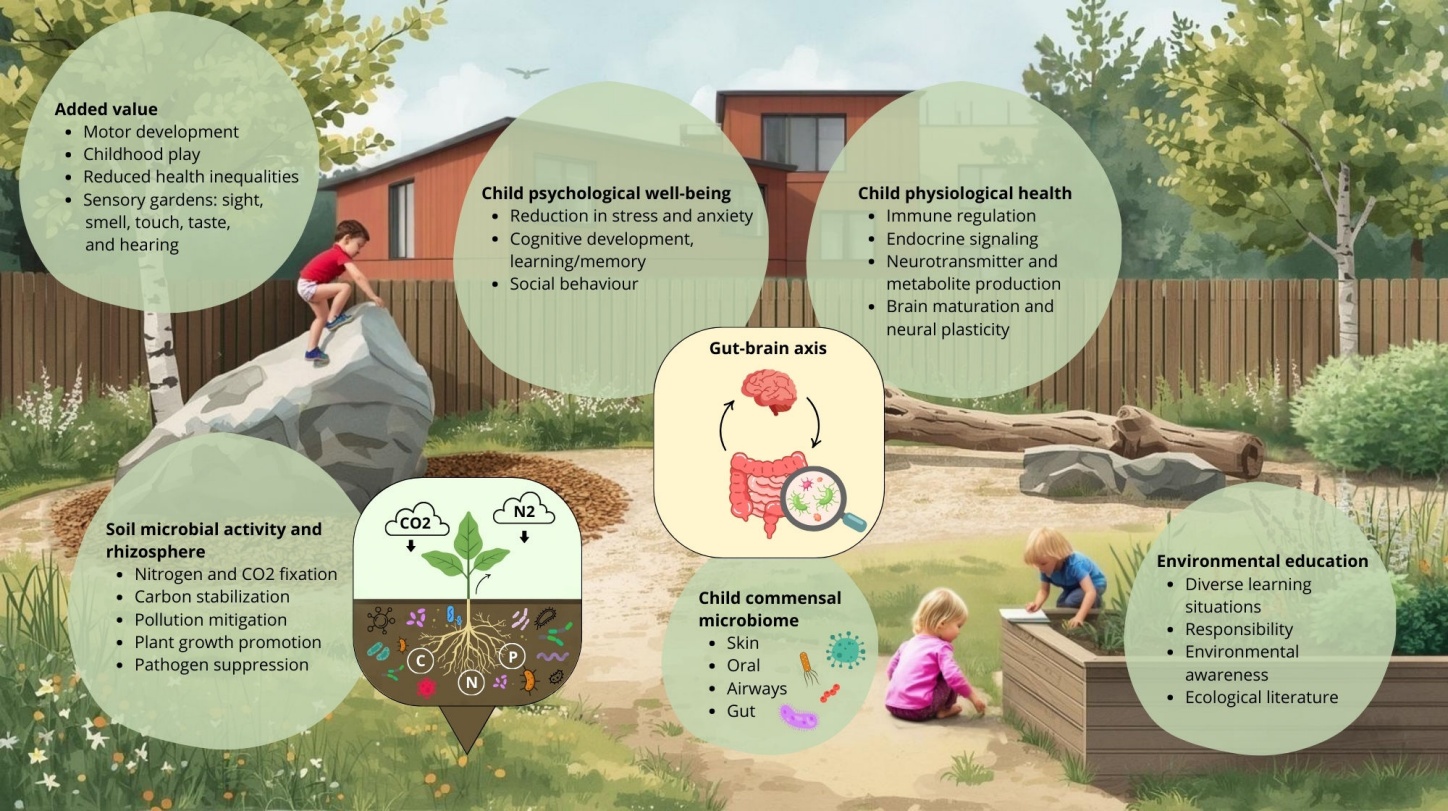
Conceptual framework of playgrounds as multifunctional microbiome interfaces within a planetary health perspective. Playgrounds are reframed as ecological systems that support children’s commensal microbiomes and psychological and physiological health, while soil microbial activity in the rhizosphere simultaneously delivers key ecosystem services. Together, these functions illustrate how playgrounds can serve as living nodes where child health and planetary health are interconnected, with added benefits that include environmental education, reduced health disparities, motor development, and sensory-rich discovery gardens for play.

In this minireview, we synthesize emerging research at the intersection of microbial ecology, child health and well-being, and sustainable urban planning, with a focus on playgrounds as multifunctional microbiome interfaces within a planetary health framework (Fig. 1). We ask how may spatial heterogeneity of urban microbiomes influence child health strategies? What strategies could reduce the spread of antibiotic resistance genes? How can playgrounds be optimized to promote microbial diversity and microbial processes, focusing on microbial activity related to pollutant mitigation? How can such environments simultaneously support the development of human commensal microbiomes, immune regulation, and endocrine signaling in children? We focus on environmental features that facilitate exposure to beneficial environmental microbiota and explore how soil-centric, biodiverse playgrounds may act as ecological and physiological modulators. We highlight the promise of multi-omic systems biology approaches to disentangle these complex host–microbe–environment interactions. We also discuss the potential of microbiome-informed playgrounds to enhance well-being, physical activity, learning, connectedness to nature, and environmental awareness.

## SPATIAL HETEROGENEITY OF URBAN MICROBIOMES

Cities have often been considered as poor in biodiversity, but studies have also shown that urban greenspace soils host high microbial richness, often exceeding that of nearby natural ecosystems such as forests (26, 27). Within cities, soil microbial and especially bacterial richness has been linked to urbanization intensity, with more densely populated areas showing higher diversity (28), possibly driven by higher soil pH (29–31). From this perspective, the availability of microbial diversity within cities is not inherently limited. For example, soil sampled within New York City’s Central Park hosted microbial richness comparable to samples collected across natural ecosystems spanning multiple biomes and continents (32). However, such studies have focused mostly on the subset of urban land that is green and permeable. When the broader urban matrix is considered, there are more impervious surfaces (e.g., concrete and asphalt), reduced vegetation, and limited organic soil exposure. Thus, the average resident in highly urbanized areas is exposed to lower environmental microbial diversity than individuals in suburban or rural settings (33, 34). Indeed, studies have shown that urban soil microbiomes are globally homogenized, showing similar community composition across cities and continents, whereas natural ecosystems display much greater variability across regions and climatic gradients (26, 35). At the same time, urbanized soils contain higher relative abundances of potential human pathogens and allergens, as well as antibiotic-resistance genes (ARGs) (7, 26, 34, 36–39).

A comprehensive approach has been proposed to mitigate ARGs in urban soils, combining control, environmental management, and surveillance (40). Key strategies include improved management of wastewater, hospital effluents, and pet or urban-agriculture waste. In addition, managing soils and vegetation to support resilient soil microbial communities, deploying molecular surveillance tools, and intentionally designing urban infrastructure can all help reduce ARG persistence (40). Expanding antibiotic stewardship beyond clinical settings and increasing awareness among urban planners and the public are also essential to reduce urban ARG reservoirs and associated health risks (40).

The aerobiome, the microbial community of outdoor air, is a key exposure pathway for human health and declines in abundance and diversity with urbanization (41). Recent studies highlight the role of urban green spaces in shaping aerobiomes (42, 43). Research from Shanghai demonstrates that neighborhoods with higher vegetation cover and less gray infrastructure show reduced indoor transfer of airborne ARGs, particularly during warmer seasons (43). This suggests that urban biodiversity can act as a natural microbial filter, shaping which outdoor microbes enter homes through the air. Complementary evidence from Zhao et al. (42) indicates that the phyllosphere is a major source of beneficial airborne microbes, while soils may help trap or neutralize harmful microbes, thereby limiting their aerosolization. Biodiversity also plays a critical role: new gardens with low biodiversity, especially in colder seasons, were linked to a higher presence of microbes associated with respiratory diseases compared to old gardens with high biodiversity (44). Overall, these findings suggest that well-established, biodiverse, and year-round urban green spaces can shape the urban aerobiome, thereby reducing exposure to ARGs and respiratory pathogens.

Climate and seasonality further modulate urban microbiomes. Warm and humid climates tend to support higher microbial growth, fungal diversity, and a richer aerobiome, while cold or arid climates typically exhibit lower microbial biomass and more dust-associated taxa (33, 37, 45, 46). Seasonal dynamics are pronounced, with winter periods in northern climates showing reduced microbial richness and increased representation of opportunistic pathogens, compared to summertime (33). In humid subtropical climates, short-term weather patterns such as wind speed and humidity strongly shape the aerobiome through resuspension and aerosolization processes (47).

Together, these findings suggest that while urban soil microbiomes are globally homogenized (26, 31, 35), spatial heterogeneity may still exist within and between urban areas. Local factors such as vegetation structure, land-use intensity, pollution, and regional climate may shape both soil and airborne microbial diversity and function at neighborhood and city scales. Given this complexity, spatial heterogeneity must be explicitly incorporated into models of microbiomes and child health outcomes. Vegetation type and plant species richness can override abiotic soil traits in shaping urban microbial communities (48, 49). Incorporating spatial variability and climate considerations into urban planning and public health strategies may, therefore, enable targeted, equitable interventions, such as prioritizing biodiverse green spaces in highly urbanized neighborhoods, to support child immune development and overall health.

## PLAYGROUNDS AS MICROBIAL INTERFACES

### Playground microbiomes and pollution

Playground soil microbiomes are shaped by substrate type and surrounding vegetation (7, 50). Therefore, selecting appropriate soil substrates and vegetation represents an opportunity to enhance microbial diversity and activity, thereby strengthening ecosystem functions. In modern urban environments, artificial turf and rubber surfacing are increasingly used in playgrounds for aesthetics, safety, and accessibility. While numerous studies have examined the negative environmental and health impacts of these materials (51–53), little is known about their influence on microbiomes, both environmental microbiomes in the playground and the commensal microbiomes of children who play on these surfaces.

To date, only a handful of studies have addressed microbial communities on artificial playground surfaces. One study found that artificial rubber surfaces harbored more potentially pathogenic bacteria than natural substrates (54). When comparing equally dry and harsh habitats at playgrounds, artificial turf versus natural rocks, microbial communities on turf exhibited a low number of indicator species but higher network connectivity and complexity, indicating potentially increased interdependence and reduced community stability (55). This pattern is often associated with environmental stress, such as drought (48, 49, 56). Similarly, the bacteriome exhibited lower diversity and abundance on turf (55), further supporting the interpretation that the artificial turf microbiome may be less resilient compared to natural surfaces.

Soininen et al. (57) proposed a framework for “immunomodulatory urban greening,” using forestry and agricultural side streams to enrich urban soils with health-associated microbial taxa. In this experimental study, moss, conifer needles, and reed were added as amendments to lawn soil, which led to an increased relative abundance of Proteobacterial classes (57). In addition, alder litter and conifer needles supported the presence of *Mycobacterium* spp. These results indicate shifts in microbial communities that, in earlier studies, have been associated with positive changes in immune regulation (2, 8, 58, 59). Although these findings are promising, real-world evidence from playground settings is still scarce. Future studies are needed to determine whether enhancing soil microbial diversity in playgrounds can deliver concrete health benefits for children, while also improving broader ecosystem services such as pollutant mitigation.

Playgrounds in urban areas are subjected to various potentially harmful pollutants that can be carried in from the surrounding landscape by atmospheric deposition, effluents, and solid wastes (60). Within playgrounds, pollutants can also emerge from synthetic surface materials and play equipment (51, 61, 62) or pre-existing soil contamination (63). The primary concern in controlling health risks from pollutants would be to reduce further inputs from the environment and playground infrastructure by replacing the synthetic materials with natural materials.

Urban pollutants, such as polycyclic aromatic hydrocarbons (PAHs) from traffic emissions and residential wood burning, are well known to disrupt immune and endocrine signaling and cause other negative health effects in children (64, 65). Beyond their direct toxicity, PAHs also shape soil microbial communities, often altering the same microbial taxa that have been linked to immune function and chronic non-communicable disease in humans (66, 67). Furthermore, soil and air PAH levels at playgrounds have been associated with skin microbiota of children and microbial functions in the gut related to endocrine signaling (63). Therefore, the Altered Environmental Microbiome Hypothesis proposes that both biodiversity loss and urban pollutants modify urban microbiomes, with cascading effects on human commensal microbiota and health (66). Importantly, urban playgrounds and other green spaces could be designed or managed to enhance microbial diversity and activity, thereby supporting pollutant mitigation while promoting healthier microbial exposures for children.

Soil processes driven by microorganisms can provide tools to mitigate pollution on site through immobilization, detoxification, and/or degradation of pollutants. Roslund et al. (67) demonstrated that by selecting appropriate landscaping and gardening soil materials, we can promote microbial activity relevant to pollutant mitigation. In practice, bioremediation potential by native soil microbial communities can be stimulated with organic amendments, such as compost, biochar, and organic mulches and living plant cover (68–70). Understanding how plant–microbe interactions within the rhizosphere further enhance pollution mitigation is highly valuable. Plants can stimulate microbial communities that degrade or transform pollutants, forming the basis of phytoremediation (69). Multi-omic approaches, such as metagenomics and metatranscriptomics, now enable detailed characterization of these rhizosphere-driven biodegradation pathways and the functional responses of plant-microbe systems to environmental pollutants (69).

### Plant-microbe synergy: rhizosphere, phyllosphere, and endosphere

**Fig 2:**
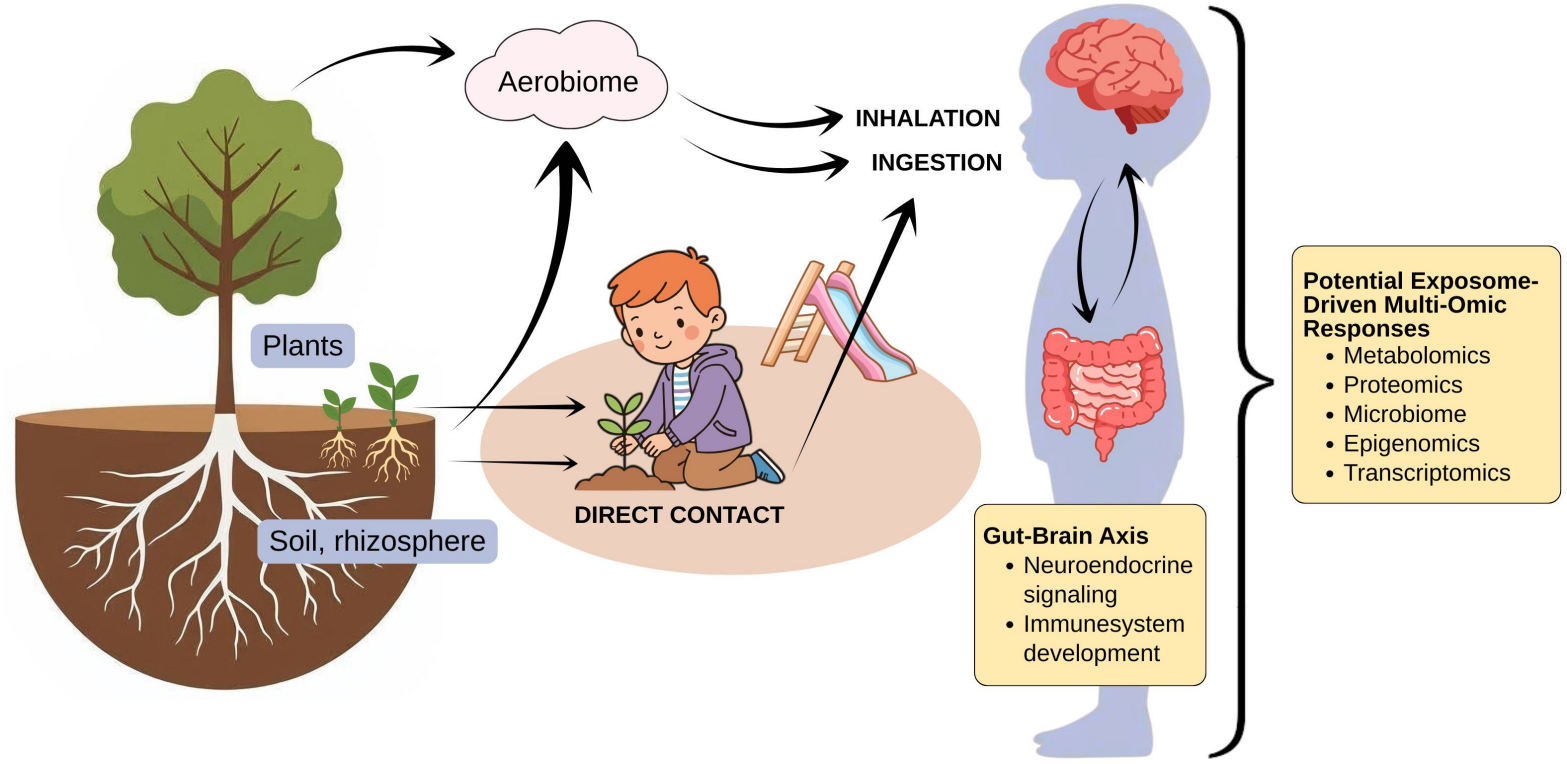
Microbial exposure routes at playgrounds and potential exposome-driven multi-omic responses in children. Children are exposed to environmental microbiomes through direct contact, inhalation, and ingestion, with implications for gut–brain axis-related processes. Soil and plant characteristics influence the aerobiome by shaping airborne microbial composition, diversity, and abundance. These exposures are associated with responses across multiple biological layers in children, including microbiome, metabolomic, proteomic, transcriptomic, and epigenomic profiles.

Plants live in tight interactions with diverse microbial consortia that affect plant survival, health, and reproduction. Plant-related microbes have the potential to influence the human microbiome through direct physical touch (71), aerobiome (72), and consumption of plant parts (17) (Fig. 2). Residential greenness has been observed to correlate positively with the diversity of the human microbiota (18), and the yard vegetation is associated with gut microbiota of the garden owner (16).

The rhizosphere, comprising an integrated network of plant roots, soil, and a microbial community of bacteria, fungi, nematodes, and viruses, is directly influenced by the plant root metabolites. This communication is bidirectional: microbes can, in turn, promote plant growth and stress resilience (14). This bidirectional communication makes the rhizosphere a hotspot for biological activity with complex interactions between the plants and soil organisms. Understanding, predicting, and managing the rhizosphere can help optimize soil microbial diversity, plant–microbe interactions, and soil processes in urban playgrounds. This knowledge enables the design of resilient green spaces that support plant health, enhance ecosystem services such as soil carbon storage, and improve responses to environmental stressors like pollution, compaction, and climate variability (73). A majority of the microbial assembly in the rhizosphere is determined by soil characteristics, while a smaller but important proportion is determined by the plant genetics (sometimes referred to as the “core rhizosphere microbiota”), regardless of the rhizosphere environment (74). In accordance with this, soil bacterial diversity in urban green space soils has been observed to be positively associated with plant species richness (75). Mycorrhizal fungi are the most studied rhizosphere organisms, and more than 80% of all plants are expected to rely on mycorrhizae to obtain nutrients, especially phosphorus (76). In addition to increased nutrient availability, microbial activity in the rhizosphere can outcompete pathogens and influence plant hormonal signaling (14). Although the role of rhizosphere microbiota in suppressing plant pathogens is well established (77, 78), their potential to suppress human pathogens in urban green spaces remains comparatively underexplored. This gap is particularly relevant in playground contexts where children frequently interact with soil and surface environments. Cooley et al. (79, 80) found that epiphytic bacteria can suppress human pathogens such as *Salmonella enterica* and *Escherichia coli* in the rhizosphere and phyllosphere. In another study, Yang et al. (81) observed that a diverse rhizosphere microbiome can constrain the prevalence of human and animal pathogens in soil. These findings underline even further the importance of diverse rhizosphere and soil microbiota in children’s play environments.

The phyllosphere, particularly the leaves and buds, provides an extensive microbial habitat due to the wide surface area of global vegetation. Similar to the rhizosphere, it hosts diverse communities of bacteria, fungi, archaea, algae, and viruses, all of which play important roles in plant–microbe interactions. These microbes engage in relationships such as mutualism and antagonism, leading to complex plant-associated communities (82). Symbiotic interactions in the phyllosphere contribute to plant growth, enhance resilience by protecting against pathogens through biocontrol, and influence gene functions (83). Collectively, these processes benefit not only plant health and productivity but also ecosystem stability and services. In addition to symbiotic plant–microbe interactions, previous studies have reported that plants and their microbiota have an important role in shaping airborne and household dust microbiota and possibly directly affect health-relevant microbial exposure (15, 72, 84, 85). Different plant species, and even cultivars, can host distinct microbiomes (86). Thus, microbially informed plant selection in urban green spaces has been proposed as a strategy to optimize human microbiota and planetary health (86). This could enhance health-promoting environmental exposures through urban design. However, the method has not been tested in large-scale intervention studies.

In the endosphere, the internal tissues of plants, microbes play critical roles in nutrient uptake, synthesize phytohormones, and enhance tolerance to environmental stresses (87). They also contribute to pathogen protection by competing with or inhibiting harmful organisms and supporting plant resistance (87–89). The endosphere also has growing relevance for human health and the microbiome. Many endophytic bacteria and fungi are capable of producing bioactive metabolites (90). These metabolites include antimicrobials, antioxidants, and immunomodulatory compounds that can indirectly affect human health through dietary intake of plants but also directly shape the gut microbiome (17, 90–92). Evidence from animal models indicates that some endophytic microbes can persist through the food chain, indicating that plants may act as carriers of beneficial microbes that colonize the gut (93). This colonization can be either temporary or permanent. Regular interaction with plants containing diverse endophytic communities could therefore contribute to maintaining or restoring gut microbiota (93–95). Although further confirmation in human models is needed, evidence suggests that endophytic microbiota, particularly in raw plants, fruits, and vegetables grown in the wild or within home gardens, may play an important role in shaping bacterial communities relevant to human health (96, 97). Based on these findings, cultivating more edible plants in early childhood environments could provide both beneficial microbial exposure and additional educational value to children.

### Nature exposure, child-microbe synergy, and health

Early-life and even prenatal microbial exposures are now recognized as pivotal in shaping the developing immune system and promoting tolerogenic immune functions, especially during the first 1,000 days of life (3, 98, 99). These processes influence a broad spectrum of immune-mediated and inflammatory conditions, including rheumatoid arthritis, psoriasis, atopic dermatitis, type 1 diabetes, asthma, allergic rhinitis, and certain neurological disorders (3, 98, 100). The emerging “nature exposure–microbial diversity–health” axis is increasingly supported by epidemiological, experimental, and intervention studies (5). This underscores the role of diverse environmental microbiota in shaping children’s commensal microbiota and health.

Living in proximity to natural green spaces has been associated with protective effects against multiple inhalant atopic sensitizations, potentially mediated by increased gut Actinobacteria diversity (101). Similarly, farm environments—rich in environmental microbial diversity—have been linked to lower prevalence of asthma (102–105). More recently, agricultural environments have been associated with a reduced risk of type 1 diabetes (106). Notably, children with similar genetic risk for type 1 diabetes but living in urbanized Finnish Karelia have a sixfold higher incidence compared to those in neighboring Russian Karelia (107). This may be related to the higher exposure to microbial diversity in Russia (108).

Evidence linking children’s residential greenness to allergic and respiratory outcomes remains mixed and sometimes contradictory. While studies suggest protective effects, such as decreased risk of asthma and allergic sensitization among children raised in greener environments (109, 110), some studies have linked surrounding greenness to increased risk of asthma in children (111–113). Indeed, in pan-European analyses, greenness was positively associated with allergic rhinitis in two cohorts but inversely in the other two birth cohorts (114). It seems that not all forms of greenness appear equally beneficial. For instance, proximity to coniferous forests was associated with increased risk of wheezing, asthma, and allergic rhinitis (115), and a higher abundance of local fauna species has been linked to greater allergy risk (116). Similarly, residential proximity to forests was protective against obesity and sedentary behavior but unrelated to asthma, whereas living near parks was associated with higher asthma risk (112). Taken together, these findings highlight that “greenness” is not a uniform exposure, and its health impacts depend on vegetation type, landscape context, and potentially other co-occurring environmental factors. Importantly, without incorporating insights from microbiome research and multi-omic approaches to understand how environmental exposures affect children’s regulatory systems, such findings are likely to remain inconclusive. None of the studies directly examined environmental microbiomes, meaning that differences in microbial exposure patterns, alongside pollen and chemical emissions, may underlie the observed inconsistencies. Seasonality may also be an important factor. Early-life exposure to green spaces during spring has been associated with an increased risk of developing allergic rhinitis into young adulthood, whereas summer exposure appears to reduce this risk (117). Future research that integrates multi-omic systems biology approaches with environmental characteristics (e.g., environmental microbial communities, vegetation type, and land use) and urban design is crucial for clarifying when and how green spaces support children’s health, and for informing evidence-based playground and green space planning.

#### Experimental intervention studies

Experimental studies that incorporate microbial perspectives provide further mechanistic insights into how environmental exposures influence children’s commensal microbial communities and regulatory systems. Schoolyard biodiversity has been shown to drive short-term recovery of disturbed skin microbiota in children (118). Randomized controlled trials indicate that regular play in biodiverse natural environments can alter gut microbial composition, stabilize gut serotonin levels, and lower stress indicators such as cortisol (119, 120). A biodiversity intervention study demonstrated that environmental greening of playgrounds may enhance commensal microbiota and support immune regulation (8). Notably, the skin microbiota of children playing in more natural playgrounds exhibited increased Gammaproteobacterial diversity. This diversity correlated with higher levels of TGF-β, reduced plasma concentrations of pro-inflammatory IL-17A, and an increased proportion of regulatory T cells (Tregs) that help maintain immune tolerance (8). Another study similarly linked higher skin Gammaproteobacterial diversity of children to nature exposure and elevated levels of anti-inflammatory IL-10 and reduced risk of atopy (2). Biodiversity intervention effects on skin and saliva microbiota, including higher Gammaproteobacterial diversity, persisted for at least 2 years and included reductions in opportunistic pathogens such as *Haemophilus parainfluenzae*, *Streptococcus* spp., and *Veillonella* spp. (7).

Interestingly, placebo-controlled studies have confirmed that exposure to microbial diversity can modulate systemic immune markers (6, 19). In one trial, children playing in sandboxes filled with microbially enriched sand—compared with visually similar but low-microbial-content sand—exhibited a higher IL-10 to IL-17A ratio (6). This indicates a shift toward an anti-inflammatory immune profile. Similar effects were observed in adults cultivating plants indoors in urban environments (19).

The gut microbiome is well known to modulate the endocrine system and exert broad influences on host behavior, metabolism and appetite, growth, reproduction, and immunity (121, 122). At least six observational cohort studies suggest links between green living environments and children’s gut microbiota (101, 102, 123–126). Five intervention studies have attempted to test these associations more directly (6–8, 119, 120). Of these, three involved modifying childcare center playgrounds to increase microbial exposure (6–8). Roslund et al. (8) observed changes in the gut only among children at greened playgrounds with high microbial diversity. These changes were observed in the Ruminococcaceae family, including well-known butyrate producers. This change in gut microbiota may reduce inflammation, because butyrate is a short-chain fatty acid that supports intestinal barrier integrity and regulates immune responses (127). Comparable shifts in Ruminococcaceae have also been reported in other nature-exposure studies with adults and animals (16, 128).

Collectively, these findings suggest that biodiverse environmental contact can shape both skin and gut microbiomes, with downstream effects on immune regulation. This positions environmental microbiota as an underutilized tool in preventive child health strategies. However, long-term prospective studies are still needed to determine whether microbially oriented nature exposure at playgrounds can prevent immune-mediated diseases in children.

## WELL-BEING, COGNITIVE, AND SOCIAL BENEFITS OF GREEN PLAYGROUNDS— POTENTIAL LINKS TO GUT MICROBIOTA?

Green play environments are well known for supporting children’s health and well-being, physical activity, learning, cognitive development, and ecological literacy (129–132). Fostering soil literacy from an early age may strengthen understanding of the interconnections between soil, human, and planetary health (133). Evidence indicates that green playgrounds increase diverse play opportunities and foster creative, unorganized “free” play (132, 134, 135) that is more inclusive for children (136, 137). Studies also show benefits for physical activity (138–140) and motor development (141), alongside reductions in physiological stress and improvements in self-reported psychological well-being (142) and perceived restorativeness (143–146). Cognitive and learning-related gains include enhanced attentiveness (140, 143, 147), cognitive development (148), expanded learning opportunities (134, 149, 150), and improved academic attainment (151). Children with attention deficit hyperactivity disorder and autism spectrum disorder may experience improvements in attention, emotional regulation, and social functioning through regular engagement with nature (152, 153). Additional reported benefits of green playgrounds include greater prosocial behavior (138, 154–156), stronger connectedness to nature (146), and the development of positive environmental relationships (134, 150, 157).

Beyond these observable behavioral and cognitive benefits, emerging evidence suggests that some of these effects may be mediated through changes in the human gut microbiome. The gut microbiota is known to influence stress responses and mental health via the gut–brain axis, a bidirectional communication system between the gut and the brain that helps maintain overall physiological homeostasis (23). Gut microbes and their metabolic products support nutrient absorption, protect against pathogens, and regulate inflammation and insulin sensitivity (158). Gut microbes can influence how hypothalamic–pituitary–adrenal axis responds to chronic stress-related disorders, including anxiety and depression, which in turn shapes children’s psychological outcomes, immune balance, and metabolic health (127). Importantly, the microbiome–gut–brain axis is known to influence brain development, neural plasticity, cognition, and behavior during infancy and early childhood (159).

It is suggested that altering the gut microbiota may offer a promising therapeutic strategy for addressing abnormalities in brain development (158). However, little is known about the link between exposure to biodiverse natural environments—such as through play in soil- and plant-rich playgrounds—the gut microbiome, and the causal effect on child psychological outcomes and development via the gut–brain axis. Sobko et al. (120) found that increasing children’s engagement in nature-based play altered gut microbiota composition and fecal serotonin levels and reduced perceived stress, although the functional significance of the gut microbial changes for psychosocial outcomes remains unclear. Similarly, Puhakka et al. (134) demonstrated that greening playgrounds increases bacterial richness in the play environment while also promoting well-being, and over the long term, this intervention has been shown to shape children’s oral and gut microbiota (7). However, the causal relationship between nature exposure, microbial changes, and psychological outcomes remains unclear. Therefore, future research is needed to clarify how microbial changes induced by nature exposure contribute to children’s psychological functioning, including stress regulation, emotional well-being, and cognitive performance.

## MULTI-OMIC SYSTEMS APPROACHES IN MICROBIOME–CHILD HEALTH RESEARCH

Many different omic data layers can be analyzed along with the microbiome, such as genomics, transcriptomics, epigenomics, proteomics, and metabolomics (160). Multi-omics approaches are valuable to connect what microbes are present, what they are doing, and how the child’s body responds. The early-life exposome is the totality of environmental, behavioral, and biological exposures that interact with a child’s molecular systems and child development to shape health trajectories (161). Each omics layer captures a different dimension of biology, and by combining them, we can understand the early life exposome’s impact.

Multi-omic analyses in mouse models have shown that air-pollution exposure during growth stages disrupts metabolic crosstalk along the gut–brain axis, leading to neuronal damage potentially mediated through lipid-metabolism dysregulation and inflammation (162). Multi-omics study by Stratakis et al. (163) with human cohorts shows a similar trend; environmental pollutants are important risk factors for childhood obesity and metabolic dysfunction. Although Stratakis et al. (163) included the surrounding green space among the prenatal determinants examined, it did not reach the stability threshold and was therefore not selected as a predictor of the multi-omics cluster profiles (163). In infants, multi-omic studies have demonstrated that environmental risk factors early in life, such as cesarean section, shift gut microbiota of infants at risk of celiac disease (164). These shifts in gut microbiota and metabolomes indicate immune dysfunction and inflammation. Whereas infants without risk factors showed higher abundance of *Bacteroides uniformis* and 3-3-hydroxyphenylpropionic acid, coupled with decreasing lipoic acid and methane metabolism pathways (164). These microbial and metabolic signatures indicate enhanced anti-inflammatory and immunomodulatory activity during early development. Thus, these findings highlight the exposome’s critical role in shaping metabolic and immune health.

A large multi-omic exposome study of 1,301 mother–child pairs demonstrated how early-life environmental exposures were linked to widespread molecular changes during childhood (165). Exposome consisted of a wide range of chemical, outdoor, social, and lifestyle exposures assessed in pregnancy and childhood, together with multi-omics profiles (methylome, transcriptome, proteins, and metabolites) measured in children. In childhood, environmental exposures elicited molecular responses across all omics layers, with the serum metabolome showing the strongest signal (165). Exposure to natural spaces in pregnancy showed few associations with child miRNA and methylome, whereas built environment in childhood was associated with shifts in the proteome. However, natural spaces and built environments showed fewer associations compared to other exposomes, such as maternal smoking during pregnancy and child exposure to toxic chemical compounds (165). This suggests that toxic compounds strongly shape children’s internal omics profiles, whereas the influence of green environments remains less certain. Nevertheless, evaluating whether increased microbial exposure through green elements could mitigate toxin-induced disruptions by reshaping the microbiome and its downstream metabolic pathways would be highly valuable.

A longitudinal personal-exposome study indicated that environmental microbial exposures may indeed form part of the exposome that shapes internal multi-omics and health outcomes (166). Gao et al. (166) estimated chemical and biological components and linked them to gut microbiome, proteome, and metabolome. The authors demonstrate significant correlations between microbial exposures and gut microbiota or immune/proteomic signatures (166). Among the proteome–exposome interactions, Gao et al. (166) identified 11 “highest-degree” exposome factors that each correlated with more than 22 proteins. Nine of these were microbial genera, and two were chemicals. The high-degree microbial genera were primarily fungi and were mostly positively correlated with the proteins. However, the study does not explicitly analyze exposures to green space or environmental microbiota from natural environments, so direct evidence on nature‐derived microbial exposures remains lacking.

Collectively, these findings illustrate how multi-omic systems approaches can uncover links between early exposures, microbiome development, and child health trajectories. However, no published studies to date appear to apply a multi-omic approach to the child’s play environment or to exposure to natural microbial communities, nor to examine the resulting effects on the child’s commensal microbiome and health. Such integrative future studies are needed to establish causal links, not just associations, between green living/play environment, microbial exposure, and child health outcomes, such as immune and endocrine signaling as well as cognitive benefits.

Multi-omics could be a useful tool to link children’s health to the soil microbiome. Although previous studies indicate associations between soil-derived microbial taxa (such as Gammaproteobacteria on the skin) and children’s health outcomes (2, 6–8, 167), there are still no standardized indicators directly linking children’s health to the soil microbiome. Potential indicators could integrate soil microbial diversity indices, the presence of beneficial or pathogenic taxa, antibiotic resistance genes, and exposure biomarkers in children. These biomarkers could be, for example, microbial metabolites, soil-associated bacterial taxa, such as *Mycobacterium*, *Streptomyces*, or certain Gammaproteobacteria (58, 167, 168). In addition, functional genes or metabolic profiles, e.g., genes related to the degradation of soil organic matter or plant-associated metabolites, are possible indicators linking soil microbiomes to child health (57, 169). Developing such indicators would require longitudinal, multi-omic studies that combine environmental, microbiological, and health data. However, establishing universally accepted indicators may not be feasible due to numerous confounding factors, including regional and climatic differences in soil microbial composition, variability in land use and exposure pathways, differences in children’s genetics, diet, and lifestyle, and socioeconomic or urbanization gradients that shape both microbial exposure and health outcomes. These complexities suggest that context-specific or regional indicators might be more realistic than universal ones.

## RETHINKING THE IMPLEMENTATION OF PLAYGROUNDS

Nowadays, playground standards mandate the installation of impact-attenuating surfaces to reduce the severity of falls. Yet, the required depth and extent of soil removal for compliant surfacing often severs the connection between children and living soil, eliminating microhabitats and reducing opportunities for microbial exposure and natural sensory experiences (136). Efforts to minimize soil compaction and limit excavation are, therefore, critical for sustaining soil microbial activity, but in practice, they may conflict with strict regulatory compliance.

Protecting root zones and soil integrity during construction is essential, yet clearance standards often require removal of trees and understory vegetation, undermining both ecological preservation and children’s opportunities for beneficial microbial contact. New planting areas may enhance aesthetics and provide play or learning opportunities, but they rarely compensate for the loss of mature vegetation and continuous soil systems.

Research in recreation ecology shows that recurrent trampling leads to soil compaction, vegetation loss, and long-term ecological change (170). Therefore, appropriate playground sizing, circulation management, and resilient design are essential to limiting the degradation of soil and vegetation (136, 171). In playgrounds, soil and vegetation together form microhabitats that regulate carbon sequestration, nutrient cycling, water infiltration, and microclimate (172). Vegetation sustains soil life by maintaining structure, supporting microbial activity, and moderating environmental stress (172). In the context of playground implementation, prioritizing soil-based vegetation and living substrates over artificial or isolated green elements is essential for sustaining microbial diversity and ecosystem services. Key considerations for designing playgrounds as microbial interfaces within the planetary health framework are outlined in Table 1, spanning environmental sustainability, child health and development, educational dimensions, and safety.

**TABLE 1:** Considerations in the implementation of playgrounds in the planetary health concept and microbial interface

I. Environmental sustainability and ecosystem health
1. Native and biodiverse vegetation
Use local, non-invasive plant species.
Choose low-allergen species (e.g., avoid high-pollen trees like birch and ash).
Promote plant diversity to support soil health, pollinators, and microbial richness (75, 86).
Avoid plants with toxic berries or thorns in areas accessible to children.
Include layers: trees, shrubs, ground cover for structural and microbial complexity (173).
2. Air quality mitigation
Plant pollution-buffering vegetation (e.g., trees and hedges) between roads and play areas (174).
Optimize airflow in design to minimize trapping of pollutants at breathing height (174).
3. Microbiome-supportive soils
Avoid synthetic turf; instead, use living soils, mulch, or natural substrates (55, 57).
Incorporate leaf litter, logs, compost, and decomposing wood to support beneficial soil microbes (57).
Promote contact with natural materials (soil, plants, and wood) to support commensal microbial diversity (7, 8).
Avoid over-sanitized surfaces and excessive use of antimicrobial products in natural zones, e.g., pesticides, etc.
4. Water management
Use permeable surfaces to reduce runoff.
Consider rain gardens, bioswales, and native wetland plants to manage excess water and improve local water cycles.
II. Child health and developmental needs
5. Physical activity and motor skill development
Include diverse terrains: hills, logs, rocks, and natural climbing features.
Provide spaces for running, jumping, climbing, and balancing to support gross motor skills (141).
6. Sensory and cognitive engagement
Use varied textures, smells, and colors (e.g., aromatic herbs, bark, and sand).
Include open-ended elements like loose parts, mud kitchens, and digging zones for imaginative play.
Create quiet zones for reflection, sensory regulation, or children with neurodiverse needs (152, 153).
7. Social and inclusive play
Design for different age groups and abilities.
Provide accessible paths and equipment for children with mobility challenges.
Include group-friendly areas to encourage cooperative play.
III. Educational and psychological dimensions
8. Environmental literacy and stewardship
Include interactive features like edible gardens, insect hotels, bird feeders, or compost stations.
Soil in a jar with different plants and minerals handled and assembled by children is one example of an educational tool that can be used to get their hands dirty with beneficial microbial exposure and learn about the terrestrial biogeochemical cycle with endless scope for interdisciplinary learning (12).
Use signage or storytelling elements to explain ecological functions (e.g., pollination and decomposition) (9).
As the sustainability of future societies depends on the knowledge and actions of younger generations, fostering soil literacy from an early age can strengthen understanding of the interconnections between soil, human, and planetary health (133).
Use curricular plants for education purposes.
9. Mental well-being and stress reduction
Incorporate green, shaded, and restorative zones for quiet play and calm.
Nature exposure is linked to lower stress, better mood, and attention regulation in children (120, 129, 131, 156).
Green playgrounds afford well-being, play, and environmental relationships (134).
IV. Safety, maintenance, and community integration
10. Safe and non-toxic materials
Use natural, durable, non-toxic materials (e.g., untreated wood).
Avoid artificial turf and plastic-heavy designs (51–53).
11. Community involvement and local context
Co-design with children, caregivers, educators, and local ecological experts.
Respect cultural, social, and climatic context.
12. Climate resilience and seasonal functionality
Design for local climate conditions (e.g., shade in hot climates, drainage during monsoons).
Include year-round features that adapt to seasonal changes.

### Priority principles for ecologically informed playground design

- Align with urban-scale ecological objectives: design solutions should be guided by broader ecological networks, specifically supporting either canopy-covered systems, open-habitat networks, or water-retention and stormwater systems, depending on the local context and strategic priorities.
- Preserve existing vegetation and soils: maintaining established vegetation ensures the existence of associated soil and microbial communities during and after construction.
- Maximize total vegetation cover: both retained and newly planted vegetation should be prioritized, as this enhances aboveground biodiversity and strengthens the continuity of belowground living-soil networks.
- Favor locally prepared soil substrates over peat-based materials: on-site soil construction methods contribute to sustainability while supporting native soil microbial communities.
- Promote structurally diverse planting: incorporate multilayered vegetation aboveground and heterogeneous root architectures belowground to enhance resilience and ecological function.
- Integrate organic material into design: employ deadwood, surface litter, and natural play elements as functional components of the playground, contributing to habitat quality and children’s experiential engagement.
- Design for microhabitat diversity: use varied topography and surface forms to generate a mosaic of microhabitats, supporting biodiversity and ecological learning opportunities.

## CONCLUDING REMARKS

By viewing playgrounds as living microbiome interfaces, we can reframe their value—not simply as places for play, but as ecological systems that deliver key ecosystem services such as biodiversity support and pollutant mitigation. Available literature suggests that these spaces have the potential to support children’s commensal microbiome and immune and endocrine regulatory systems, mental well-being, and environmental learning. This perspective aligns with the planetary health framework that links the health of human populations with the integrity of the natural systems on which they depend.

Current literature indicates that loss of microbial diversity in the environment and the child’s body may reverberate across multiple dimensions of child health. This loss may contribute to immune-mediated and allergic diseases, neurodevelopmental and metabolic dysfunction, increased exposure to pathogens and antibiotic-resistance genes, as well as to cognitive, behavioral, and mental health problems.

Current knowledge about how modern urban playgrounds shape microbiomes—both the environmental communities present in soils and vegetation and the commensal microbiomes of children who play there—remains limited. There is a lack of studies utilizing high-throughput approaches, such as metagenomics, metabolomics, and proteomics, to investigate ecological and health-related impacts of environmental features or characterize host–microbiome interactions relevant to immune development, endocrine signaling, and psychological outcomes. Ecological processes and human health outcomes are rarely studied together within a planetary health framework. Notably, no studies to date have demonstrated causal links between exposure to microbial communities in nature, the gut microbiome of children, and the resulting psychological outcomes, such as stress regulation and cognitive performance, via the gut–brain axis. Long-term trials are needed to assess whether microbially oriented nature interventions can support healthy immune and endocrine regulatory systems, thereby eventually reducing the incidence of immune-mediated diseases, as well as endocrine- and stress-related disorders. Addressing these gaps is essential for developing evidence-based strategies for designing playgrounds and other urban green spaces that support both environmental and child health. Framing such approaches as preventive public health strategies within a planetary health perspective highlights their potential to reduce the long-term burden of disease, while simultaneously fostering resilience through healthier microbiome–host interactions and more sustainable urban ecosystems.
